# Impact of the COVID-19 pandemic on depression, anxiety, loneliness, and satisfaction in the German general population: a longitudinal analysis

**DOI:** 10.1007/s00127-022-02311-0

**Published:** 2022-06-09

**Authors:** Nora Hettich, Theresa M. Entringer, Hannes Kroeger, Peter Schmidt, Ana N. Tibubos, Elmar Braehler, Manfred E. Beutel

**Affiliations:** 1 Department for Psychosomatic Medicine and Psychotherapy, University Medical Center of the Johannes Gutenberg University Mainz, Mainz, Germany; 2 German Institute for Economic Research (DIW Berlin), Berlin, Germany

**Keywords:** COVID-19 pandemic, General population, Mental health, Depression and anxiety symptoms, Life and health satisfaction, Loneliness

## Abstract

**Purpose:**

Cross-sectional studies found high levels of depression and anxiety symptoms, and loneliness during the first wave of the COVID-19 pandemic. Reported increases were lower in longitudinal population-based findings. Studies including positive outcomes are rare. This study analyzed changes in mental health symptoms, loneliness, and satisfaction.

**Methods:**

Respondents of the German Socio-Economic Panel (*N* = 6038) were surveyed pre-pandemic (2017/2019) and during the first (June 2020) and second wave (January and February 2021) of the pandemic. Self-report screeners assessed depression and anxiety symptoms, loneliness, life and health satisfaction. Difference scores were analysed using ANCOVAs focusing on time, gender, age groups.

**Results:**

Depression and anxiety symptoms and health satisfaction increased from pre-pandemic to the first wave, but declined in the second pandemic wave. Loneliness increased and life satisfaction decreased during the first and the second wave of the pandemic. Young adults and women reported more distress and loneliness, even after controlling for pre-pandemic scores, education, and income. All effects remained stable when controlling for self-reported previous diagnosis of depression or region of residence.

**Conclusion:**

Increases and decreases in mental health symptoms and health satisfaction showed little variation. Of concern are the strong increases of loneliness and decreased life satisfaction being important targets for interventions. Main risk factors are young age and female gender.

**Supplementary Information:**

The online version contains supplementary material available at 10.1007/s00127-022-02311-0.

## Introduction

Mental health has become a major concern facing the global threat of the COVID-19 pandemic and the drastic changes of the living and working conditions of the general population due to containment measures. Meta-analyses have reported high prevalence rates of depression and anxiety symptoms during the first COVID-19 wave based on numerous cross-sectional studies relying on online convenience samples (e.g., anxiety rates between 25% [1] and 38% [2], rates of depression symptoms of 34% [3]). A large British online panel with over 70,000 participants starting from March 2020 reported decreasing anxiety and depression symptoms during the first 20 weeks following the initial lockdown [4]. Based on the three-item UCLA Loneliness Scale, different degrees of loneliness were reported with 32.5% feeling sometimes and 18.3% often lonely exceeding figures from previous panels (28.6%, respectively 8.5% [5]). On the other hand, Kivi and colleagues [6] found no increase in loneliness among older participants. Fewer studies indicated lowered life satisfaction [7, 8]. However, estimates of online surveys are likely to be biased [9], as participants are mostly those feeling strongly affected by the pandemic or wanting to contribute to its investigation and pre-pandemic scores were lacking [10, 11].

A number of longitudinal cohort studies compared pre-pandemic scores to those in the first wave of the pandemic. A recent meta-analysis that included 65 studies showed an increase in mental health symptoms (particularly in depression symptoms) during March and April, 2020 and a decline of symptoms during May and July, 2020 [12]. The UK Household Longitudinal Study reported an increase of clinically relevant distress from 18.9% in 2018/2019 to 27.3% in April 2020 (GHQ12 [13]). Despite the presence of considerable COVID-19-related worries, a Swedish longitudinal study found stable life satisfaction and increased self-rated health in older adults during the first wave of the pandemic [6]. Based on over 110,000 participants, the German National Cohort [14] reported a small increase of clinically relevant depression (PHQ-9: from 6.4 to 8.8%) and anxiety (GAD-7: from 4.3 to 5.7%) for participants younger than 60. Compared with pre-pandemic levels, subjective health status, as assessed by the first question of the Short Form Health Questionnaire (SF-12), improved in 32% of the sample and deteriorated in 12% of the sample. For the UK, Daly, Sutin, and Robertson [15] reported an increase of the proportion of participants with high levels of anxiety symptoms (GAD-2) in April 2020, followed by a decline in May, and stable scores through December.

Previous studies have also indicated considerable heterogeneity concerning vulnerable groups. Risk factors for distress were young age, female gender, and low income [4, 14–16]. Findings regarding other social determinants were contradictory, low education [4] and unemployment were risk factors in many studies [1], whereas an English Study identified employment and higher education as risk factors for distress [13, 15]. The inclusion of prior mental health diagnoses as a possible risk factor also appears to be important, as previous findings have been conflicting. A review and meta-analysis of longitudinal cohort studies found no evidence of increased symptom burden in participants with prior mental illness [12]. In contrast, an English longitudinal study of the general population found greater increases in anxiety and depression symptoms among participants with prior mental health diagnoses [4]. Studies comparing psychiatric patients with healthy controls reported the same effect [17–19].

This paper contributes to the important issue of how recurrent waves of the COVID-19 pandemic affect the mental health of the general population over time and who is most affected by the pandemic concerning mental distress. In contrast to previous studies comparing scores between different measurement time points, we used the changes in scores from before to the pandemic and between different pandemic waves. The paper analyses how depression and anxiety symptoms, loneliness, and life and health satisfaction changed among men and women of different age groups during the first and second wave of the COVID-19 pandemic in Germany. By identifying risk factors, the paper enables the planning and implementation of specific public health measurements.

## Methods

The Socio-Economic Panel (SOEP) is the largest German household panel with about 30,000 randomly selected participants in 15,000 households running since 1984 and including all regions of Germany. It is a multidisciplinary, multi-purpose study that is carried out every year independently of specific research projects that use or intend to use the SOEP data. It is conducted in accordance with the Declaration of Helsinki and all participants give their informed consent prior to data collection. As the current study analyzed anonymized data, an ethics approval was not required. Detailed information on ethical clearance and informed consent of the SOEP and the SOEP-CoV study can be found on the websites of the German Institute for Economic Research (DIW), Berlin (https://www.diw.de/soep and https://www.soep-cov.de).

The study “socio-economic factors and consequences of the spread of coronavirus in Germany” (SOEP-CoV) assessed a random subsample of 11,999 households from April 1 to June 28, 2020 and again from January 18 to February 15, 2021 by computer-assisted telephone interviews (CATI). During the first survey assessment in 2020, there was an average of 1384 new SARS-CoV-2 infections per day across Germany, with a peak of 6029 cases. At the end of the assessment, 193,499 persons were infected with SARS-CoV-2 and 8957 persons had died in Germany. In the second assessment in 2021, the average number of new infections in Germany per day was 9890 with a peak of 19,385. At the end of this phase, 286,812 persons in Germany were infected and 18,657 had died. In 2020, a total of 6694 regular SOEP respondents answered questions about their health, attitudes toward the first few months of the COVID-19 pandemic in Germany, and their economic and family situation [10]. In 2021, a total of 6038 persons were re-interviewed during the second wave of the pandemic. The questionnaires can be accessed at https://www.soep-cov.de/Methodik/Methodology/index.php/. To maintain the representativeness of the sample, a weighting procedure was used (detailed description in supplement).

### Measures

Sociodemographic information, such as age, gender, education, household income, region of residence, and information about previous medical diagnosis of depression, was either available from prior waves of the survey or assessed during the first- or second-wave interviews. Education was stratified as primary, secondary, and tertiary based on the highest level of education or vocational training according to the Comparative Analysis of Social Mobility in Industrial Nations (CASMIN). The equivalized household income covers all disposable monthly household incomes, including all types of after-tax and transfer income. Differences in household size are considered by equivalization, i.e., divided by the square root of the number of household members [20]. A previous diagnosis of depression was assessed in 2017 and 2019 by asking: “Has a doctor diagnosed a depressive disorder in the last 2 years?”.

Self-report questionnaires assessed depression and anxiety symptoms (PHQ-4) in 2019, 2020, and 2021. The two-item questionnaire PHQ-2 measures anhedonia (“Little interest or pleasure in doing things.”) and depressed mood (“Feeling down, depressed, or hopeless.”) over the past 2 weeks [21]. Its rating scale ranges from 0 to 6. Scores of 3 and above indicate a depressive disorder with a sensitivity of 79% and a specificity of 86% [22]. The PHQ-2 shows an internal consistency of α = 0.75 [23]. In this sample, the internal consistency was α = 0.68.

Anxiety symptoms were measured with the two-item GAD-2 questionnaire. It assesses whether one suffered from feeling nervous, anxious, or on the edge in the past 2 weeks and was unable to stop or control rumination [21]. The range of scores is from 0 to 6, and scores of 3 and above indicate an anxiety disorder (e.g., generalized anxiety, panic disorder, social phobia) with a sensitivity of 65%, a specificity of 88% [24], and an internal consistency of α = 0.82 [23]. In this sample, the internal consistency was α = 0.68.

Loneliness was assessed in 2017, 2020, and 2021 by the German version of the three-item UCLA Loneliness Scale [25]. The questions are: “How often… (1) … do you feel that you lack companionship? (2) … do you feel isolated from others? (3) … do you feel left out?” Participants rated the questions without time restriction in 2017. In 2020 and 2021, they were asked to rate the questions referring to the past 2 weeks. Responses were scored on a five-point Likert scale (never, hardly ever, sometimes, often, very often). The sum score provided a loneliness scale ranging from 0 to 12, with a higher score indicating higher levels of loneliness. There is no established cut-off value for a high level of loneliness in the literature. For the descriptive analysis of this study, we set a cut-off score of 7 to indicate a high level of loneliness. A participant must score at least one item with the second highest score and consequently, frequently experiences at least one aspect of loneliness. This indicates that lack of companionship, isolation, or feeling left out, as indicators of loneliness, is a regular theme in the participant’s daily life. The German version of the three-item UCLA Loneliness scales shows an internal consistency of α = 0.79 [26]. In this sample, the internal consistency was α = 0.71.

Satisfaction with life and with health were determined in 2019, 2020, and 2021 by the valid [27] single items „How satisfied are you currently with your life?” and “How satisfied are you with your health?”, respectively. Both items were rated on a scale from 0 (“completely dissatisfied”) to 10 (“completely satisfied”). In this study, dissatisfaction is indicated by scores from 0 to 3, medium satisfaction by scores from 4 to 6, and satisfaction by scores from 7 to 10.

### Statistical analysis

Respondents of the SOEP were interviewed before the onset of the pandemic and twice during the pandemic. Difference scores for depression and anxiety symptoms, loneliness, and life and health satisfaction between 2019 (2017 for loneliness) and 2020 as well as between 2020 and 2021 served as dependent variables. Because the analyses focused on repeated measures of these difference scores, categorical group variables, and control for social contextual factors, three-way ANCOVAs with post hoc analyses (Tukey method) were conducted. These three-way ANCOVAs used ANOVAs with the within-sample factor time (repeated measures) and the categorical between-samples factors sex and age groups (categorical group variables) to estimate mean differences between these three factors and their interactions which is equivalent to regression models with dummy variables [28]. Furthermore, regression models for the metric covariates pre-pandemic outcomes, education, and equivalized income (social contextual factors) were used to control for the effects of these variables [29]. In additional ANCOVAs, we controlled for pre-pandemic outcomes, for a prior self-reported depression diagnosis, and for region of residence. Effect sizes correspond to partial Eta^2^ (*η*_p_^2^ ≥ 0.01 small, *η*_p_^2^ ≥ 0.06 medium, and *η*_p_^2^ ≥ 0.14 large effect). Significance for statistical tests was set at *p* < 0.05 (two-sided). All analyses were conducted using R version 4.0.3 (packages: tidyvers [30], afe [31], lsmeans [32]).

## Results

### Study participants

From April 1 to June 28, 2020 during the first wave of the pandemic in Germany and from January 18 to February 15, 2021 during the second wave, 6038 participants of the regular SOEP-sample were interviewed. As shown in Table 1, the total sample included 6038 participants with a mean age of 55 years (age range 18–101 years). Sixty-one percent were female, 32.5% had tertiary education, and 80.4% of the participants lived in West Germany. The mean equivalized household income was 2257€ (*SD* = 2321.07) per month. About 10% of the participants reported a medical diagnosis of depression in the last 2 years before 2019 and 2017 respectively.

**Table 1:** Sample characteristics in 2021 (*N* = 6,038)

Age in years (*N* = 6030)	M	SD
Average age	55.2	15.7
Age groups (*N* = 6030)	*N*	*%*
18–29	353	5.9
30–49	1832	30.4
50–69	2595	43.0
70–101	1250	20.7
Gender^1^ (*N* = 6031)		
Male	2364	39.2
Female	3667	60.8
Education (CASMIN classification) (*N* = 5913)		
Primary	1922	32.5
Secondary	3624	61.3
Tertiary	367	6.2
Equivalized income in Euro^2^ (*N* = 5432)	M	SD
Average income per month	2257.0	2321.1
Household size (min. 1; max. 10)	2.51	1.36
Region of residence (*N* = 6031)	*N*	*%*
West Germany	4850	80.4
East Germany	1181	19.6
Previous depression diagnosis^3^ (*N* = 5867)		
No	5273	89.9
Yes	594	10.1

^1^Diverse gender was not a selection category; ^2^Median = 2,000.0 Euro; ^3^ in 2017 and 2019 participants were asked about a medical diagnosis of depression during the last 2 years; sociodemographic information (age, gender, education, region of residence) was available from the 2019 survey

### Descriptive course of mental distress, loneliness, and satisfaction

Table 2 shows the proportion of participants who reported scores above the cut-offs for depression and anxiety symptoms and loneliness and the distributions of life and health satisfaction before and during the first and the second wave of the pandemic. Clinically relevant symptoms of depression and anxiety were most commonly reported during the first wave (PHQ-2: 13.9%; GAD-2: 8.6%). During the second wave, this percentage decreased slightly (PHQ-2: 12.1%; GAD-2: 7.9%) but did not reach pre-pandemic levels (PHQ-2: 9.7%; GAD-2: 6.8%). Loneliness scores above the cut-off increased more than fourfold during the first (from 6.1 to 27.4%) and showed a further increase during the second wave of the pandemic (31.8%). Life satisfaction decreased slightly during the first COVID-19 wave (from 82.2 to 80.8% satisfied) and continued to do so during the second (73.5%). Particularly, the percentage of participants being neither dissatisfied nor satisfied with their life increased (25.2%) while the proportion of participants being unsatisfied align with the pre-pandemic level in 2021 (1.2%). Health satisfaction increased during the first (72.9%) and declined during the second wave (67.9%) but remained above the pre-pandemic level (62.3%). Proportions of participants reporting dissatisfaction with health or neither dissatisfaction nor satisfaction declined during the first and increased during the second wave remaining below pre-pandemic levels.

**Table 2:** Percentages of participants above the cut-off scores for depression and anxiety symptoms and loneliness and different levels of satisfaction (dissatisfied 0–3; neither dissatisfied nor satisfied 4–6; satisfied 7–10) with life and health

	Depressiveness (PHQ-2)	Anxiety (GAD-2)	Loneliness (UCLA)	Life satisfaction (0–10)			Health satisfaction (0–10)		
	Cut-off 3 95% (CI)	Cut-off 3 95% (CI)	Cut-off 7 95% (CI)	Range 0–3 95% (CI)	Range 4–6 95% (CI)	Range 7–10 95% (CI)	Range 0–3 95% (CI)	Range 4–6 95% (CI)	Range 7–10 95% (CI)
2017/2019^1^	9.6 (8.9; 10.4)	6.7 (6.1; 7.4)	5.9 (5.3; 6.5)	1.2 (1.0; 1.5)	16.5 (15.6; 17.5)	82.2 (81.2; 83.2)	4.4 (3.9; 4.9)	33.3 (32.1; 34.5)	62.3 (61.1; 63.6)
2020	13.8 (12.9; 14.7)	8.6 (7.9; 9.3)	27.1 (26.0; 28.3)	0.5 (0.3; 0.7)	18.7 (17.7; 19.7)	80.8 (79.8; 81.8)	2.2 (1.9; 2.6)	24.9 (23.8; 26.0)	72.9 (71.8; 74.0)
2021	12.0 (11.2; 12.9)	7.9 (7.2; 8.6)	31.6 (30.5; 32.9)	1.2 (0.9; 1.5)	25.2 (24.1; 26.4)	73.5 (72.4; 74.6)	3.2 (2.8; 3.7)	28.8 (27.7; 30.0)	67.9 (66.7; 69.1)

^1^Loneliness was assessed in 2017 while the other outcomes were assessed in 2019; PHQ-2 = Patient Health Questionnaire-2, scores range from 0 to 6; GAD-2 = Generalized Anxiety Disorder-2, scores range from 0 to 6; UCLA = Three-Item Loneliness Scale, scores range from 0 to 12; life satisfaction = 1-item-question, scores range from 0 to 10; health satisfaction = 1-item-question, scores range from 0 to 10

### Analyses of changes in mental distress, loneliness, and satisfaction

Difference scores of depression and anxiety symptoms, loneliness, and life and health satisfaction from pre-pandemic to pandemic (2020) and from the first to the second wave (2021) were analysed controlling for pre-pandemic outcome scores, equivalized income, and level of education. Table 3 shows the analyses of covariance by time (2020, 2021), gender (male, female), and age groups (18–29, 30–49, 50–69, 70–101).

**Table 3:** Analyses of covariance with difference scores of depression and anxiety symptoms, loneliness, and life and health satisfaction by time (2020, 2021), gender (male, female), and age groups (18–29, 30–49, 50–69, 70–101) controlling for pre-pandemic scores, level of education and logarithmized, equivalized income

	Depression symptoms (PHQ-2) df = 4296			Anxiety symptoms (GAD-2) df = 4296			Loneliness (UCLA) df = 4049			Life satisfaction df = 4296			Health satisfaction df = 4296		
	*F*	*p*	*η*_p_^2^	*F*	*p*	*η*_p_^2^	*F*	*p*	*η*_p_^2^	*F*	*p*	*η*_p_^2^	*F*	*p*	*η*_p_^2^
Time	70.05	< 0.001***	0.016	58.82	< 0.001***	0.014	219.11	< 0.001***	0.051	28.05	< 0.001***	0.006	125.24	< 0.001***	0.028
Gender	36.14	< 0.001***	0.008	33.23	< 0.001***	0.008	39.40	< 0.001***	0.010	7.44	0.006**	0.002	2.08	0.149	< 0.001
Age	17.52	< 0.001***	0.012	13.86	< 0.001***	0.010	6.31	< 0.001***	0.005	6.90	< 0.001***	0.005	18.23	< 0.001***	0.013
Time × gender	0.08	0.775	< 0.001	5.65	0.017*	0.001	13.62	< 0.001***	0.003	0.22	0.635	< 0.001	5.55	0.019*	0.001
Time × age	1.10	0.347	< 0.001	0.51	0.676	< 0.001	0.44	0.721	< 0.001	0.46	0.707	< 0.001	14.57	< 0.001***	0.010
Age × gender	0.89	0.444	< 0.001	0.84	0.474	< 0.001	1.17	0.051	< 0.001	2.12	0.096	0.001	0.70	0.555	< 0.001
Time × gender × age	1.10	0.346	< 0.001	2.24	0.082	0.002	0.98	0.399	< 0.001	2.46	0.061	0.002	1.20	0.308	< 0.001

Covariates: pre-pandemic outcome score (2017 for loneliness and 2019 for depression and anxiety symptoms and life and health satisfaction), level of education, and equalized income; *p-value < 0.05; **p-value < 0.01; ***p-value < 0.001; PHQ-2 = Patient Health Questionnaire-2; GAD-2 = Generalized Anxiety Disorder-2; UCLA = Three-Item Loneliness Scale; Life and health satisfaction = One-item-question each

**Fig. 1:**
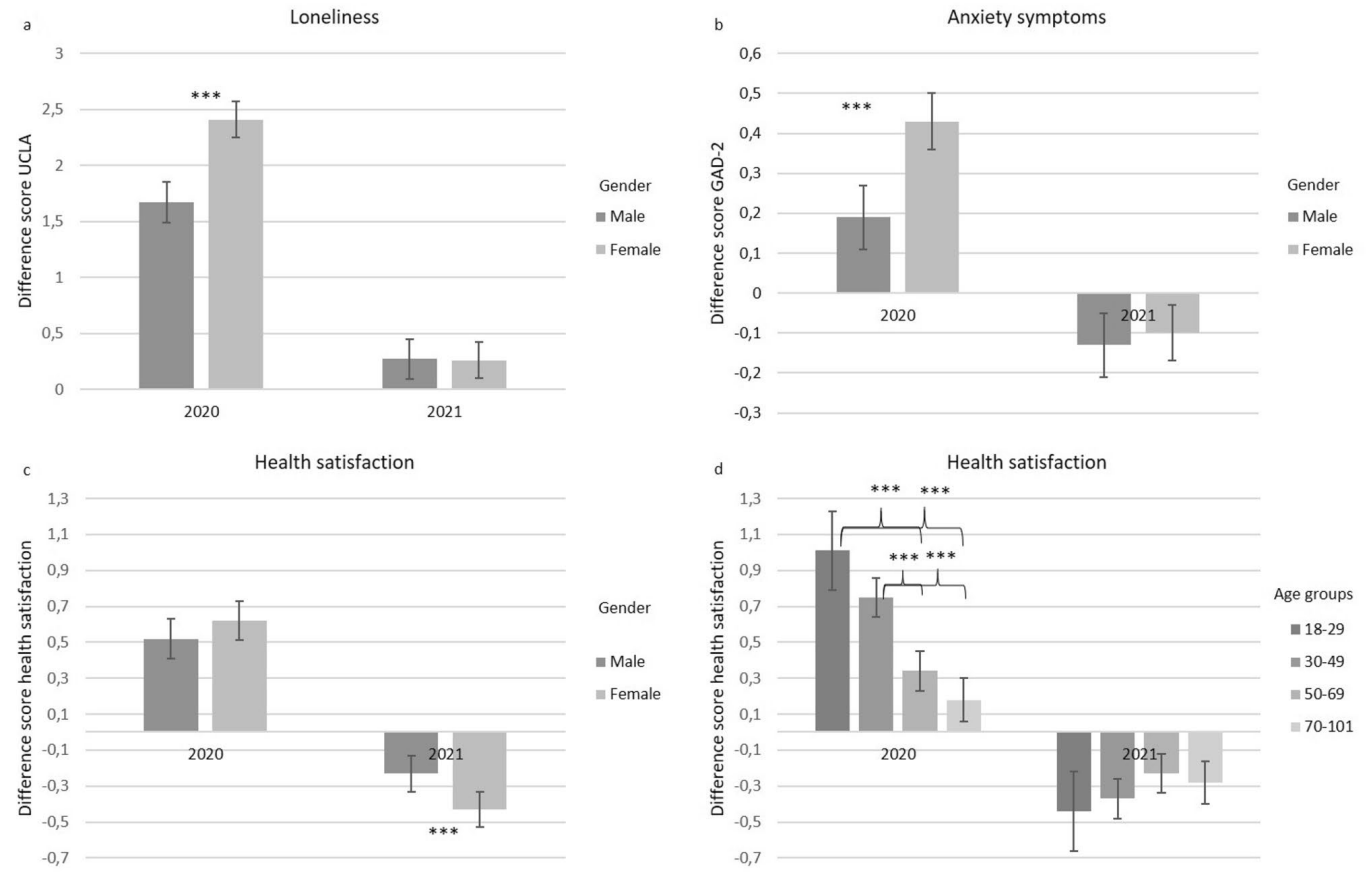
Changes in outcome variables indicating significant interaction effects with time: **a** interaction effect of time and gender for loneliness, **b** interaction effect of time and gender for anxiety, **c** interaction effect of time and gender for health satisfaction, **d** interaction effect of time and age groups for health satisfaction

The interaction effects (time × gender) for anxiety symptoms, loneliness, and health satisfaction are presented graphically in Fig. 1a–c. With respect to anxiety symptoms and loneliness, changes were higher in women only in the first wave of the pandemic (Fig. 1a and b). The strength of decreases of anxiety symptoms during the second wave did not differ between women and men. For health satisfaction, the interaction effect was reverse. During the first wave, men and women did not differ in terms of increases in health satisfaction. However, the decrease in health satisfaction during the second wave was stronger in women (Fig. 1c). The interaction effect time × age for health satisfaction indicates that during the first wave, younger individuals (18–29 and 30–49) differed in increases in health satisfaction from older participants (50–69 and 70–101), which was not true for decreases in the second wave (Fig. 1d).

**Fig. 2:**
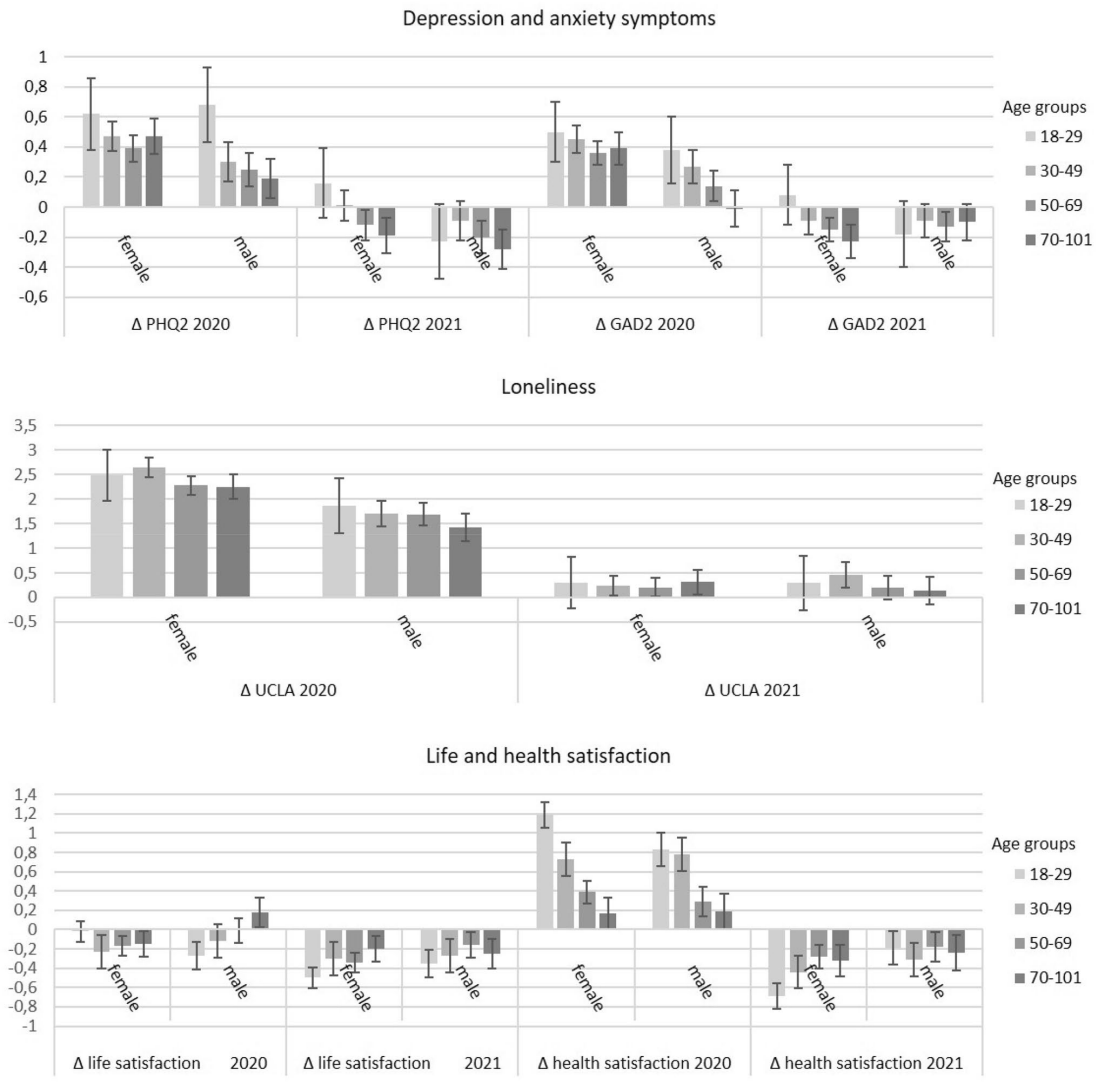
Difference scores of depression and anxiety symptoms, loneliness, and life and health satisfaction between pre-pandemic years 2017/2019 and the first wave of the pandemic in 2020 as well as between the first and the second wave of the pandemic in 2021 grouped by gender and age groups. PHQ-2 = Patient Health Questionnaire-2; GAD-2 = Generalized Anxiety Disorder-2; UCLA = Three-Item Loneliness Scale; Life and health satisfaction = One-item-question each

Figure 2 shows changes from baseline scores stratified by age groups and gender during the first and second wave of the pandemic. A main effect of time was found throughout, indicating that changes from the pre-pandemic period to the first wave were different from changes between the first and the second wave of the pandemic (small effect sizes). Overall, depression and anxiety symptoms and health satisfaction increased during the first but decreased during the second wave. Loneliness increased strongly during the first and less so during the second wave. Life satisfaction decreased slightly during the first wave and decreased even more during the second wave. A main effect of gender was found with women reporting higher increases in depression symptoms and higher decreases in life satisfaction for both time periods. Considering age, participants in the youngest age group (18–29) showed the highest increases in depression and anxiety symptoms, loneliness, and health satisfaction, and the highest decreases in life satisfaction (Fig. 2). For the main effects of age groups, post hoc tests showed that anxiety symptoms increased mostly in participants aged 18–29 and 30–49. Depression symptoms increased mostly in the youngest age group (18–29). Participants aged 30–49 reported higher increases for loneliness, compared with individuals in the older age groups (50–69 and 70–101). The lowest decreases in life satisfaction were reported by older participants, which differed from persons aged 18 to 29 and 30 to 49. All effects were stable when controlling for pre-pandemic outcomes and self-reported prior medical diagnosis of depression. They also remained stable when controlling for pre-pandemic outcomes and region of residence (Tables in the supplement).

## Discussion

Unlike current meta-analyses based on cross-sectional studies [1, 3], cohort studies [13], and longitudinal studies [12] focusing on one wave of the pandemic, in a German longitudinal household panel, we found small increases of depression and anxiety symptoms during the first wave of the pandemic in 2020 as compared to the previous year and a decrease of those symptom during the second wave in 2021. The initial increase of symptoms is consistent with another large-scale German cohort study [14] and a meta-analysis of international longitudinal studies that reported small effects sizes [33]. However, we further found slightly lower depression and anxiety symptoms in the second wave of the pandemic in 2021, despite the introduction of new lockdown measures in the last 2 weeks of 2020.

Our results also indicate considerable heterogeneity in changes in scores by age groups and gender when controlling for pre-pandemic scores, level of education, and equivalized income and further still for self-reported prior medical diagnosis of depression and region of residence. Internationally, the initial focus of public health concerns was on older people who were exposed to the greatest threat by the illness and were also expected to be more distressed by the contact restrictions of the pandemic management [34]. However, consistent with emerging evidence [3, 14, 16], depression and anxiety symptoms increased especially in young participants aged 18–49 and in women. Although women showed higher increases of anxiety symptoms during the first wave of the pandemic their decreases in the second wave did not differ from men’s changes.

Consistent with a British population-based cohort [5], we did find a large increase in loneliness during the first wave of the pandemic. Unlike reported depression and anxiety symptoms, there was even a further increase during the second wave. Women in particular reported increased loneliness during the first wave, which aligned with the increase in men during the second wave. This finding is particularly interesting given that the stability of loneliness across the lifespan has been found to be high [35] and loneliness is considered a trait-like characteristic [36]. Although the UCLA loneliness scale, used in the survey, addresses trait loneliness [37], our results showed considerable increases in loneliness from pre-pandemic to pandemic period and also from the first to the second wave of the pandemic. It is likely that the radical change in social reality during the pandemic affected the stability of loneliness, as it can be defined as a painfully experienced absence of social contact, belonging, or a sense of isolation and thus can be considered a major consequence of curbing social contacts as a central component of pandemic management. To determine potential long-term mental health consequences of the pandemic, loneliness will need to continue to be monitored after lockdown measures have ended. A previous publication was able to show that increased loneliness during the first lockdown in Germany in 2020 decreased again following relaxations of social distancing measures in the same year, i.e., loneliness behaved more state-like within this short period of time in a crisis situation [38]. It will be interesting to observe over an extended period of time whether loneliness stabilizes at current levels or returns completely to pre-crisis levels once the pandemic subsides and pandemic measures are lifted. In contrast to Kivi [6], our longitudinal data indicate that loneliness is indeed the strongest mental health response to the pandemic. This finding is highly relevant to public and mental health considerations as loneliness is known to constitute a risk for depression [39], anxiety, and suicidal ideation as well as for physical illness and premature death [40]. Reflecting the manifold restrictions of social, cultural, and leisure time activities and the burden of adjustment to pervasive changes of work, education, and childcare, we detected a decline of life satisfaction during the first and further during the second wave of the pandemic particularly in young people and women. These findings should be taken seriously, as life satisfaction is also an important predictor of (mental) health [8]. Therefore, support measures that target the particular stresses of young people and women during the pandemic should be implemented.

Intriguing is that we even found an initial increase regarding satisfaction with health, particularly in young people, during the first wave. This complements a Swedish study with an older sample [6]. In quality of life research, the satisfaction paradox denotes the fact that inner standards, values and norms may shift over the course of an illness, reducing the negative impact of objective factors in one’s life on subjective quality of life [41]. Most likely, COVID-19 has been perceived as a significant health threat continuously taking a toll of thousands of casualties suffering from severe disease or even premature death. Thus, given the fact that the majority of the population has been spared contracting an unpredictable and potentially deadly illness, health satisfaction may have initially increased rather than declined. However, the spread of the infection in the population necessitating the second lockdown may have increased the awareness of susceptibility leading to a decline of health satisfaction. This has affected women in particular, who tend to be more health conscious than men.

### Limitations

The contact restrictions of the pandemic necessitated a shift of survey mode from face-to-face in previous waves to telephone interviews during the pandemic. It is therefore possible that differences in responses before and during the pandemic were due in part to differences in survey mode (e.g., the timeframe of the loneliness scale). We used a well-established method to adjust the sample for selectivity by reverting to the large pre-pandemic data set. Although we believe this accounts for all important factors of selectivity, we cannot rule out the possibility that some degree of unobserved selectivity still exists due to previously unknown relevant factors.

We had only brief but validated screening instruments available. While the strength of our longitudinal study is the comparison to previous scores of the same participants, we acknowledge that scores for depression and anxiety symptoms, and satisfaction were obtained 1 year before the pandemic, while loneliness was assessed in 2017. Other aspects of the pandemic and the measures taken to control it, particularly changes in social life and activities, were not included in the analyses. Particularly with regard to the evolution of loneliness over time, further research should include the effects of social distancing and changes in relationships with friends, family, and members of one’s household. In addition, the effects of socioeconomic factors, such as changes in employment and income, should be examined. We were able to control for a prior diagnosis of depression but not for other mental health disorders. Our study covers a large German community sample during the first and the second wave of the pandemic. As this most likely affected only small subsamples, we did not assess stresses by contracting or witnessing infections. We cannot rule out the possibility that adaptation to the pandemic was different in other countries with higher infection and mortality rates or a temporary breakdown of the health care system due to the pandemic.

## Conclusion

Longitudinal studies are needed to understand the impact of the pandemic on mental health in the general population [10]. Our longitudinal findings suggest only small changes during the first year of the pandemic regarding the most common symptoms of depression and anxiety. The initial increase in health satisfaction, particularly among young people, may be interpreted as a shifting phenomenon in the face of the global health threat posed by the pandemic. However, the subsequent decline may also indicate the stresses of the ongoing pandemic, particularly for women. Of concern regarding future public health is the decreased life satisfaction and the large increase of loneliness. To determine mental health effects of the pandemic, it is necessary to identify the risk groups for maladjustment. Here, we found clear-cut risks for young people aged 18 to 49 and for women. A better understanding of risk profiles is an important prerequisite for targeting interventions to reduce the public mental health burden of the pandemic [42]. An important first step would be to raise awareness of the mental health burdens of the pandemic through a social marketing campaign targeting young people and women along with public health information regarding the pandemic. It would be relevant to inform these groups that depressiveness and anxiety, as well as loneliness and lack of life and health satisfaction, are relevant health conditions that should be monitored or treated as appropriate. It should be conveyed that health is not just physical illness and medical treatment. Opportunities to have loneliness and mental distress and illness treated should be given through information about counselling and mental health services to address and ameliorate these conditions. Since women usually go for annual check-ups (especially in gynaecology), this could be an additional place to implement the information as well as screenings to detect symptoms and risk levels and thereby early treatment [43]. On a more pragmatic level, the burdens of young people (e.g., social distancing and isolation) and women (e.g., childcare and workload) need to be addressed at the policy level to find solutions to overcome these specific burdens.

## Supplementary Information

Below is the link to the electronic supplementary material.

Supplementary file1 (DOCX 31 KB)

## Data Availability

The analyses are based on the German Socio-Economic Panel (SOEP), an independent scientific data infrastructure established in 1984. We, as users, cannot send the data to the journal and make them publicly available, as this is against SOEP’s statutes and would violate the EU General Data Protection Regulation (GDPR). However, this should not be a hurdle, as researchers from scientific institutions around the globe can access the data (free of costs) once they have signed a user contract. The scientific use file of the SOEP with anonymous microdata is made available free of charge to universities and research institutes for research and teaching purposes. The direct use of SOEP data is subject to the provisions of German data protection law. Therefore, signing a data distribution contract is the single precondition for working with SOEP data. The data distribution contract can be requested with a form which can be downloaded from: http://www.diw.de/documents/dokumentenarchiv/17/diw_01.c.88926.de/soep_application_contract.pdf.
