# Supplementary material for: Impact of the COVID-19 pandemic on depression, anxiety, loneliness, and satisfaction in the German general population: a longitudinal analysis
Source: Soc Psychiatry Psychiatr Epidemiol. 2022 Jun 9;57(12):2481–90. doi: 10.1007/s00127-022-02311-0 (PMC9181932; doi:10.1007/s00127-022-02311-0)
Supplement: Supplementary file 1 — Supplementary file1 (DOCX 31 KB) [file 127_2022_2311_MOESM1_ESM.docx]

Supplement

Accounting for selectivity – Weighting strategy

The initial weighting of households in the SOEP-CoV study was based on the household weight of participant’s last realized survey through 2018. Five steps followed in the weighing procedure. First, the initial weights were corrected for changes between the composition of the SOEP in 2018 and 2020. Second, corrections were made for households that were excluded from participation in the SOEP-CoV survey. Third, corrections were made for household contactability. Fourth, it was corrected for household willingness to participate and a marginal adjustment to a variety of population distributions was made completing the household-level weighing. Fifth, person weights were created for individual household members based on the household weights via a marginal adjustment and based on these weights projection factors were created for the household respondent to correct for selective (self)selection.

To improve the statistical efficiency of weighted analyses, weights were trimmed using the "weight distribution" method, which reduced variance and counteracted potential bias in weighted analyses from individual observations with large weights. Weight trimming was applied first at the household level and second at the individual level in the CATI weighting step. To compensate for sampling error and under-coverage, all weights were adjusted to marginal distributions of the German household population known from the micro census. For this purpose, the raking procedure was applied. This marginal adjustment was also performed at both the household level and the person level. For the second wave drop-out weights using extreme gradient boosting for classification were generated to account for potential differences in the probability to be reinterviewed with respect to mental health and socioeconomic and demographic characteristics. For more information on the weighting procedure visit https://www.soep-cov.de/Gewichtung/.

**Table 1.** Analyses of covariance with differences scores of depression and anxiety symptoms, loneliness, and life and health satisfaction by time (2020, 2021), gender (male, female), and age groups (18-29, 30-49, 50-69, 70-101) controlling for pre-pandemic scores and self-reported previous medical diagnosis of depression.

|  | Depression symptoms (PHQ-2)  df = 4,298 | | | Anxiety symptoms (GAD-2)  df = 4,298 | | | Loneliness (UCLA)  df = 4,051 | | | Life satisfaction  df = 4,298 | | | Health satisfaction  df = 4,298 | | |
| --- | --- | --- | --- | --- | --- | --- | --- | --- | --- | --- | --- | --- | --- | --- | --- |
|  | F | p | η_p_^2^ | F | p | η_p_^2^ | F | p | η_p_^2^ | F | p | η_p_^2^ | F | p | η_p_^2^ |
| Time | 61.73 | **<.001***** | .014 | 63.17 | **<.001***** | .014 | 143.76 | **<.001***** | .034 | 6.75 | **.009**** | .002 | 77.93 | **<.001***** | .018 |
| Gender | 36.34 | **<.001***** | .008 | 32.42 | **<.001***** | .007 | 39.55 | **<.001***** | .010 | 6.72 | **.010*** | .002 | 2.50 | .114 | <.001 |
| Age | 19.99 | **<.001***** | .014 | 15.20 | **<.001***** | .011 | 6.17 | **<.001***** | .005 | 6.72 | **<.001***** | .005 | 19.03 | **<.001***** | .013 |
| Time*gender | 0.00 | .972 | <.001 | 5.45 | **.020*** | .001 | 14.62 | **<.001***** | .004 | 0.02 | .887 | <.001 | 5.33 | **.021*** | .001 |
| Time*age | 2.00 | .112 | .001 | 0.58 | .626 | <.001 | 0.45 | .716 | <.001 | 0.59 | .620 | <.001 | 14.52 | **<.001***** | .010 |
| Age*gender | 0.97 | .408 | <.001 | 0.85 | .469 | <.001 | 1.19 | .313 | <.001 | 2.13 | .095 | .001 | 0.69 | .559 | <.001 |
| Time*gender*age | 1.31 | .269 | <.001 | 2.38 | .067 | .002 | 0.98 | .401 | <.001 | 2.02 | .109 | .001 | 1.32 | .266 | <.001 |

*Note:* covariates: pre-pandemic outcome score and self-reported previous medical diagnosis of depression; *p-value <0.05; **p-value <0.01; *** p-value < 0.001; PHQ-2 = Patient Health Questionnaire-2; GAD-2 = Generalized Anxiety Disorder-2; UCLA = Three-Item Loneliness Scale; Life and health satisfaction = One-item-question each.

**Table 2.** Analyses of covariance with differences scores of depression and anxiety symptoms, loneliness, and life and health satisfaction by time (2020, 2021), gender (male, female), and age groups (18-29, 30-49, 50-69, 70-101) controlling for pre-pandemic outcome scores and participants living in East or West Germany.

|  | Depression symptoms (PHQ-2) df = 4,298 | | | Anxiety symptoms (GAD-2)  df = 4,298 | | | Loneliness (UCLA)  df = 4,001 | | | Life satisfaction  df = 4,291 | | | Health satisfaction  df = 4,291 | | |
| --- | --- | --- | --- | --- | --- | --- | --- | --- | --- | --- | --- | --- | --- | --- | --- |
|  | F | p | η_p_^2^ | F | p | η_p_^2^ | F | p | η_p_^2^ | F | p | η_p_^2^ | F | p | η_p_^2^ |
| Time | 66.48 | **<.001***** | .015 | 49.88 | **<.001***** | .011 | 248.78 | **<.001***** | .058 | 16.03 | **<.001***** | .004 | 199.59 | **<.001***** | .044 |
| Gender | 40.15 | **<.001***** | .009 | 35.27 | **<.001***** | .008 | 40.52 | **<.001***** | .010 | 7.75 | **.005**** | .002 | 3.62 | .057 | <.001 |
| Age | 19.86 | **<.001***** | .014 | 14.79 | **<.001***** | .010 | 6.13 | **<.001***** | .005 | 7.06 | **<.001***** | .005 | 16.73 | **<.001***** | .012 |
| Time*gender | 0.00 | .960 | <.001 | 5.99 | **.014*** | .001 | 14.29 | **<.001***** | .004 | 0.05 | .815 | <.001 | 4.43 | **.035*** | .001 |
| Time*age | 1.92 | .124 | .001 | 0.53 | .659 | <.001 | 0.39 | .757 | <.001 | 0.62 | .601 | <.001 | 13.70 | **<.001***** | .009 |
| Age*gender | 0.97 | .408 | <.001 | 0.84 | .472 | <.001 | 1.15 | .329 | <.001 | 1.13 | .091 | .002 | 0.63 | .595 | <.001 |
| Time*gender*age | 1.34 | .261 | <.001 | 2.48 | .059 | .002 | 0.96 | .409 | <.001 | 1.99 | .114 | .001 | 4.58 | .268 | <.001 |

*Note:* covariates: pre-pandemic outcome score and living in East or West Germany; *p-value <0.05; **p-value <0.01; *** p-value < 0.001; PHQ-2 = Patient Health Questionnaire-2; GAD-2 = Generalized Anxiety Disorder-2; UCLA = Three-Item Loneliness Scale; Life and health satisfaction = One-item-questions each.
